# Identification of Risk Factors and Development of a Predictive Model for Postoperative Hypoglycemia among Diabetic Patients during the Perioperative Period

**DOI:** 10.1155/2023/8033101

**Published:** 2023-08-29

**Authors:** Zixuan Liu, Jing Dai, Xiaodie He, Jiaxi Li, Haixia Zhang, Cheng Ji

**Affiliations:** ^1^China Pharmaceutical University, Nanjing Drum Tower Hospital, Nanjing, China; ^2^Department of Pharmacy, The Second Affiliated Hospital of Soochow University, Sanxiang Road, No. 1055, Gusu, China; ^3^Department of Endocrinology, Endocrine and Metabolic Disease Medical Center, Nanjing Drum Tower Hospital Clinical College of Nanjing University of Chinese Medicine, Nanjing, China; ^4^Branch of National Clinical Research Centre for Metabolic Diseases, Nanjing, China; ^5^Department of Pharmacy, Nanjing Drum Tower Hospital, The Affiliated Hospital of Nanjing University Medical School, Nanjing, China

## Abstract

**Objective:**

To explore the incidence and influencing factors of postoperative hypoglycemia in diabetic patients during the perioperative period and to construct a risk prediction model for postoperative hypoglycemia.

**Methods:**

Patients with T2DM admitted to the nonendocrinology department of Nanjing Drum Tower Hospital from December 2019 to January 2022 were included as research subjects. Basic information, hospital blood glucose management methods, laboratory indicators, and surgery-related indicators were collected. A risk prediction model and scoring table for postoperative hypoglycemia in patients with perioperative diabetes mellitus were established.

**Results:**

A total of 440 patients were included, of which 109 had hypoglycemia, resulting in an incidence of postoperative hypoglycemia of 24.78%. The results show that preoperative C-peptide and operation duration were risk factors for postoperative hypoglycemia, while BMI and preoperative fasting blood glucose were protective factors.

**Conclusion:**

The model constructed in this study is a good method for evaluating the risk of postoperative hypoglycemia. The scoring table intuitively quantifies the risk of risk factors for outcome variables and has strong clinical practicability.

## 1. Introduction

In recent years, the incidence of diabetes as well as the proportion of diabetes among surgical patients has been increasing year by year. The prevalence of diabetes in China has reached 10.9%, and at least 10% to 20% of surgical patients have diabetes. This has led to the growing concern of perioperative hyperglycemia. Improper treatments of perioperative patients with diabetes can lead to various postoperative complications, among which hypoglycemia remains the most common [1]. This may be due to the severity of the primary condition causing surgery or hyperglycemia itself that may increase the risk of postoperative complications [2]. Without timely handling, hypoglycemia may contribute to serious adverse outcomes such as cardiovascular disease [3], coma, and even death [4]. The main causes of postoperative hypoglycemia among diabetic patients during perioperative period include preoperative fasting, inappropriate insulin administration, and insufficient sugar supplementation. Analysis of risk factors, as well as the prevention of postoperative hypoglycemia, should be a clinical issue worthy of consideration. However, there have been few studies reporting the risk factors for perioperative hypoglycemia in hospitalized patients with type 2 diabetes undergoing elective surgery and establishing a risk prediction model. Exploring the risk factors for perioperative hypoglycemia and developing a simple risk prediction model would help in timely detection, intervention, and prevention of perioperative hypoglycemia. Therefore, this retrospective study aimed to investigate influencing factors of postoperative hypoglycemia, construct risk prediction model, and develop hypoglycemia risk scoring system among perioperative patients with diabetes, thereby providing a reference for the reasonable perioperative management of blood glucose.

## 2. Materials and Methods

### 2.1. Study Population

A total of 440 hospitalized patients with type 2 diabetes mellitus (T2DM) undergoing elective surgery from December 2019 to January 2022 in nonendocrinology department of Nanjing Drum Tower Hospital were included.

### 2.2. Study Design

#### 2.2.1. Inclusion and Exclusion Criteria

The inclusion criteria were as follows: (1) aged 18 years and above; (2) confirmed diagnosis of T2DM; (3) clear surgical indication for elective surgery; (4) complete clinical data; (5) postoperative use of insulin to control blood glucose according to perioperative blood glucose management expert consensus [5].

Patients were excluded if (1) they had type 1 diabetes mellitus, gestational diabetes mellitus, or special type diabetes mellitus; (2) they had long-term combined use of drugs leading to blood glucose fluctuations; (3) they had long-term use of enteral and parenteral nutrition; (4) they had severe liver and kidney dysfunction; (5) they had Alzheimer's disease, brain atrophy, acute phase or sequelae of cerebrovascular disease, or cognitive impairment; and (6) they were unable to cooperate with the operation or evaluation due to comorbid shock and critical illness.

### 2.3. Data Collection

Variables were determined based on previous literature and clinical experience. Demographics included age, gender, duration of diabetes, body mass index (BMI), smoking and drinking history, family history, history of hypertension, and hospital stays. Laboratory indicators included preoperative fasting blood glucose (FBG), HbA1C, preoperative C-peptide, postprandial C-peptide, aspartate aminotransferase (AST), alanine aminotransferase (ALT), urine acid (UA), estimated glomerular filtration rate (eGFR), total cholesterol (TC), triglyceride (TG), high-density lipoprotein cholesterol (HDL-C), and low-density lipoprotein cholesterol (LDL-C). Operation-related indicators included level of surgery, incision type, operation duration, wound healing time, and intraoperative blood loss. In addition, in-hospital blood glucose management was recorded, including department self-management, endocrinology consultation management, and hospital blood glucose management mode.

### 2.4. Grouping

Eligible patients were divided into hypoglycemia group or nonhypoglycemia group according to the occurrence of postoperative hypoglycemia. In the light of diagnostic criteria of China Guidelines for the Prevention and Treatment of Type 2 Diabetes Mellitus (2020), postoperative hypoglycemia was defined as blood glucose lower than 3.9 mmol/L.

### 2.5. Statistical Analysis

Continuous variables were expressed as mean ± standard deviation (*x* ± *s*) or median and interquartile range (IQR) for normally distributed and non-normally distributed ones, respectively. Categorical variables were described as frequencies and percentages. Numerical differences between hypoglycemia group and nonhypoglycemia group were assessed by *t*-test or Mann–Whitney *U* test for continuous variables and chi-square test for categorical variables. Binary logistic regression was conducted to investigate risk factors of postoperative hypoglycemia among patients with T2DM. The threshold for significance was set at *P* < 0.05. All statistical analyses were conducted using SPSS, Version 26.0 (SPSS Inc., Chicago, IL, USA).

### 2.6. Construction and Evaluation of Risk Scoring System

The risk prediction model was established on the basis of univariate analysis and logistic regression analysis, and the risk scoring table of hypoglycemia in T2DM patients during perioperative period was built in line with the Framingham risk score model. Area under the receiver operating characteristic curve (AUC) was calculated to assess ability and accuracy of the prediction model to identify the occurrence of hypoglycemia [6]. According to the predicted probability from logistic regression analysis, receiver operating characteristic (ROC) curve was developed. The higher AUC value represents the better discrimination ability of the prediction model. Normally, AUC value is between 0.5 and 1. AUC greater than 0.7 indicates a good predictive ability of the model, while AUC greater than 0.9 indicates an excellent predictive ability of the model. Calibration can evaluate the accuracy of a disease risk model in predicting the probability of an outcome event, and it reflects the consistency between the predicted risk and the actual risk. Hosmer–Lemeshow goodness-of-fit test was used to evaluate calibration ability of the prediction model. The calibration curve was obtained after plotting a scatter diagram based on the actual observations (observed) and the model predicted value (expected) and fitting the linear trend line. The closer the calibration curve was to the standard curve, the better the calibration ability of the model was.

## 3. Results

### 3.1. General Information

A total of 440 patients were finally recruited, including 272 males and 128 females with an average age of 62.65 ± 10.70 years (ranging from 19 to 87 years). The duration of diabetes ranged from 1 to 40 years, with a mean duration of 8.51 ± 7.35 years. BMI ranged from 17.50 to 41.71 kg/m^2^, with a mean BMI of 24.95 ± 3.63 kg/m^2^. In terms of in-hospital blood glucose management, 165 received department self-management, 135 accepted endocrinology consultation management, and 140 had hospital blood glucose management. Hypoglycemia occurred in 109 (24.78%) patients, including 68 males and 41 females.

### 3.2. Univariate Analysis of Postoperative Hypoglycemia in T2DM Patients during Perioperative Period

Results of univariate analysis showed that there are no significant differences between hypoglycemia group or nonhypoglycemia group in age, gender, smoking and drinking history, family history, history of hypertension, level of surgery, incision type, intraoperative blood loss, postprandial C-peptide, AST, ALT, UA, eGFR, TC, and LDL (*P* > 0.05). However, statistical differences were found in duration of diabetes, BMI, preoperative C-peptide, HbA1C, preoperative FBG operation duration, TG, HDL, hospital stays, wound healing time, and in-hospital blood glucose management, with all *P* values less than 0.05. See Table 1 for details.

### 3.3. Multivariate Analysis of Postoperative Hypoglycemia in T2DM Patients during Perioperative Period

The occurrence of hypoglycemia was set as the dependent variable, and significant variables from univariate analysis were included as independent variables in logistic regression. BMI, operation time, preoperative C-peptide, and preoperative FBG were independent influencing factors of postoperative hypoglycemia. Specifically, operation time (OR = 1.267) and preoperative C-peptide (OR = 2.372) were risk factors, while BMI (OR = 0.894) and preoperative FBG (OR = 0.859) were protective factors. The risk of hypoglycemia increased by 0.267 times for each additional hour of surgery, and the risk in patients with preoperative C-peptide <500 pmol/L was 2.372 times higher than that in patients with preoperative C-peptide ≥500 pmol/L. The specific variable assignments and regression results are shown in Tables 2 and 3.

### 3.4. Development and Evaluation of Hypoglycemia Risk Prediction Model

According to the results of logistic regression, the final prediction model of hypoglycemia in T2DM patients was as follows: logit (P) = 1.647 + 0.237 *∗* operation time − 0.112 *∗* BMI + 0.864 *∗* preoperative C-peptide − 0.152 *∗* preoperative FBG.

#### 3.4.1. Multicollinearity Test

Variance inflation factor (VIF) of all independent risk factors and protective factors in the regression model was calculated. VIF values of BMI (VIF = 1.035), blood glucose (VIF = 1.061), operation time (VIF = 1.065), and preoperative C-peptide (VIF = 1.043) were all less than 5, suggesting no multiple collinearity among the variables.

**Figure 1:**
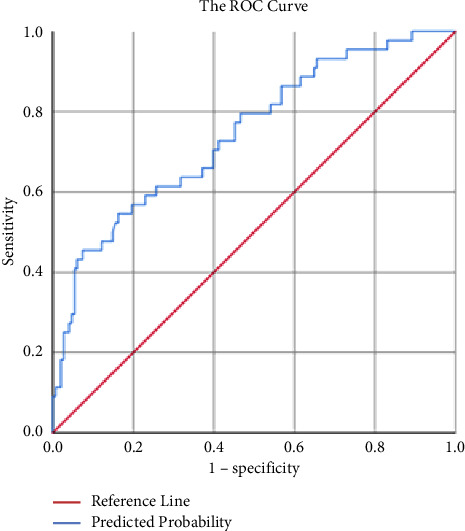
The ROC curve.

**Figure 2:**
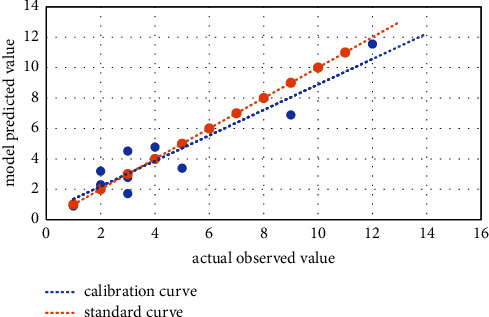
The specific calibration curve.

The ROC curve (Figure 1) was plotted based on the results of logistic multivariate analysis. The AUC was 0.745 (95% CI: 0.660–0.830) with the *P* value less than 0.05, the best cutoff value of predicted probability was 0.350, and the Youden index was 0.367 (sensitivity = 0.455 and specificity = 0.912). The above results demonstrated that the model has good ability to distinguish hypoglycemia. The Hosmer–Lemeshow test was not statistically significant (*χ*^2^ = 6.373 and *P* = 0.606), suggesting a good model fit of the regression model. See Figure 2 for the specific calibration curve. In order to intuitively reflect the logistic regression results and make the prediction model easy to be accepted clinically, a risk scoring table for hypoglycemia in perioperative T2DM patients was prepared according to the logistic regression analysis results (Table 4). The total score was obtained after adding up score of each risk factor, with the lowest score of −4 and the highest score of 10. The higher total score implied the higher risk of hypoglycemia.

Table 5 displayed the corresponding relation between the total calculated score and predicted probability. Patients were stratified according to the total score, with a score of ≤2 as the low-risk group, a score of 3–6 as the medium-risk group, and a score ≥7 as the high-risk group. The risk classification of patients could be quickly judged through the scoring table, providing a certain reference for the risk prediction of hypoglycemia in T2DM patients after operation. Disease characteristics were then substituted into the scoring table for validation. The little difference in the predicted probability value between of the scoring table and the regression model (logit (P) = 1.647 + 0.237 *∗* operation time − 0.112 *∗* BMI + 0.864 *∗* preoperative C-peptide − 0.152 *∗* preoperative FBG) suggested that accuracy of the scoring table can meet the requirements of hypoglycemia risk prediction and assessment.

The risk predicted probability value corresponding to each score was calculated according to the equation of the multivariate logistic regression model, and the calculation formula is as follows:

(1) $$\begin{array}{c} \hat{P}=\frac{1}{1+e^{\left(-\sum_{i=0}^{p}\beta_{i}X_{i}\right)}\;},\\ \overset{p}{\underset{i=0}{\sum }}\beta_{i}X_{i}\approx constant\;term+\beta_{i}*W_{ij}+B*point\;total\text{,} \end{array}$$

where *W*_*ij*_ represents the reference value of each influencing factor group and B represents the constant corresponding to 1 in the scoring tool.

(2) $$\begin{array}{c} \overset{p}{\underset{i=0}{\sum }}\beta_{i}X_{i}=1.647-0.112*25.5-0.152*5+0.237*1.2+0.864*0+0.474*point\;total\\ =-1.6846+0.474*\;point\;total\text{.} \end{array}$$

## 4. Discussion

### 4.1. Analysis of the Current Situation of Postoperative Hypoglycemia in T2DM Patients during Perioperative Period

Hypoglycemia is one of the most common perioperative complications in patients with type 2 diabetes mellitus (T2DM), with an incidence rate ranging from 5.1% to 25.3% [7]. In this study, the postoperative incidence rate of hypoglycemia in T2DM patients (24.78%) was slightly higher than previous studies, possibly due to the use of insulin after surgery [8]. Many observational studies have demonstrated a link between diabetes and adverse clinical outcomes after surgery [9–12]. Several studies have shown that patients with diabetes mellitus have an increased risk of surgical complications [13]. Any hypoglycemia after surgery is associated with increased mortality and morbidity [14]. Patients with diabetes are vulnerable to postoperative hypoglycemia due to surgical stress, impaired renal function, older age, anesthesia, postoperative fasting, and improper use of insulin [15]. However, we have not fully taken into account the differences in insulin dosage and documented them. Extensive research has been conducted in domestic and international studies on the risk factors associated with hypoglycemia in patients with type 2 diabetes mellitus (T2DM). These factors include age, duration of diabetes, body mass index (BMI), length of hospital stay, educational level, history of hypoglycemia, and previous experience with hypoglycemia education. Medication-related factors, such as the use of sulfonylureas and insulin, have also been investigated. Other factors considered include average blood glucose levels, glucose variability, glycated hemoglobin (HbA1c), renal function, triglyceride levels, diabetes complications, comorbidities, surgical procedures, and pregnancy. Lifestyle-related factors such as diet, exercise, sleep, and emotional well-being can also influence the occurrence of hypoglycemia. Among these risk factors, basal insulin dose, blood glucose variability (BG), and past history of hypoglycemic episodes have been identified as the three strongest predictors of iatrogenic hypoglycemia in hospitalized patients [16]. A recent study demonstrated a significantly lower risk of hypoglycemia if the patient is cared by an endocrinology team at all stages of surgery [17]. The implementation of our department self-management led to a significant reduction in rates of hypoglycemia in the postoperative period. In terms of blood glucose management mode, there was significant difference in incidence of postoperative hypoglycemia among department self-management (30.30%), endocrinology consultation management (26.67%), and hospital blood glucose management (16.43%), suggesting that the specialist team can contribute to reduce the occurrence of postoperative hypoglycemia. In the management process, the specialist team could formulate more reasonable and scientific insulin dosage after evaluation and multidisciplinary discussion of patient's operation condition and intraoperative blood sugar control level. Clinical pharmacists could prevent postoperative hypoglycemia through paying attention to the recovery of patients' diet after operation, giving timely feedback to doctors to adjust the dosage of insulin before meals, and communicating with patients to reduce the impact of emotional fluctuations on blood sugar, thus significantly reducing the incidence of postoperative hypoglycemia.

### 4.2. Analysis of the Influencing Factors of Postoperative Hypoglycemia in T2DM Patients during Perioperative Period

Univariate analysis revealed that there are significant differences between hypoglycemia group or nonhypoglycemia group in the duration of diabetes, BMI, preoperative C-peptide, HbA1C, preoperative FBG, operation duration, TG, HDL, hospital stays, wound healing time, and in-hospital blood glucose management. Further multivariate analysis indicated that operation time and preoperative C-peptide are risk factors while BMI and preoperative FBG are protective factors. The research conducted by Han et al. found that in patients with diabetes, a disease in the duration of ≥10 years, BMI < 18.5 kg/m^2^, and SDBG ≥ 3.0 mmol/L, and preoperative subcutaneous insulin injection is the main risk factor for perioperative hypoglycemia [18]. This study also found that the lower the BMI and preoperative blood glucose levels, the higher the likelihood of developing hypoglycemia after surgery. We found that the surgical grade and incision size in the low blood glucose group were slightly higher than those in the non-low blood glucose group. To prevent postoperative incision infections, prophylactic administration of quinolone antibiotics may be given, which can also increase the occurrence of postoperative low blood glucose.

### 4.3. Operation Time

It is worth noting that longer operation time can lead to greater risk of postoperative hypoglycemia, which has not been identified by other studies. The possible reason may be that the longer operation time reflects to some extent a more serious condition with difficult blood sugar control and prolonged postoperative fasting, thus increasing the risk of hypoglycemia after operation.

### 4.4. BMI

BMI in the hypoglycemia group was significantly lower than that in the nonhypoglycemia group (*P* = 0.007), which was consistent with the findings of Alghamdi et al. [19]. The reasons might be that there exists insulin resistance among obese or overweight patients, and the degree of insulin resistance increases in turn in nondiabetic patients, nonoverweight diabetic patients and obese diabetic patients. Insulin resistance leads to decreased sensitivity to hypoglycemic drugs and reduces the occurrence of hypoglycemia to a certain extent.

### 4.5. Preoperative FBG

Although preoperative blood glucose level has a positive effect on reducing the incidence of postoperative infection and other related complications, it cannot be ignored that strict control will increase the incidence of postoperative hypoglycemia. The results of this study showed that the preoperative FBG in the hypoglycemia group is statistically lower than that in the nonhypoglycemia group, and the preoperative FBG level is an independent factor of postoperative hypoglycemia. Since preoperative FBG reflects the blood glucose control level of the patient to a certain extent, intensive hypoglycemia will increase the risk of hypoglycemia, and postoperative patients can be more likely to hypoglycemia because of nutritional problems.

### 4.6. Preoperative C-Peptide

C-peptide can be used to assess the presence of endogenous insulin deficiency. In addition, continuous glucose monitoring (CGM) revealed that insulin-treated patients with T2DM are prone to develop hypoglycemia, many episodes of which are asymptomatic [20]. Decreased islet beta cell function is related to an increased risk of hypoglycemia. Christensen et al. and Hope et al. [21, 22] found that C-peptide levels in patients with hypoglycemic episodes are significantly lower than those in patients without hypoglycemic episodes, and patients with lower C-peptide have a more significant frequency of hypoglycemia episodes and a longer total duration of hypoglycemia. This study also showed that risk of hypoglycemia in patients with preoperative C-peptide <500 pmol/L is 2.372 times higher than that in patients with preoperative C-peptide ≥500 pmol/L.

### 4.7. Development of Hypoglycemia Risk Prediction Model in T2DM Patients during Perioperative Period

According to the result of logistic regression, risk prediction model of hypoglycemia in T2DM patients was logit (P) = 1.647 + 0.237 *∗* operation time − 0.112 *∗* BMI + 0.864 *∗* preoperative C-peptide − 0.152 *∗* preoperative FBG. A risk scoring table for hypoglycemia in perioperative T2DM patients was then developed in order to propose a more intuitive understanding of hypoglycemia for clinical workers. After adding up the score of each risk factor, the higher total score implied the higher risk of hypoglycemia. The risk factors were stratified based on the scoring table, which was helpful for clinical reference for perioperative blood glucose management. Moreover, the scoring table not only visually quantified the risk factors on outcome variables but provided more accurate clinical guidance for clinicians with simple calculation. Thus, the scoring table had strong practicability and could reflect the concept of individualized treatment.

## 5. Conclusion

This study has several advantages. First, the included patients were all T2DM patients in perioperative period, whose disease status was relatively complex with difficult postoperative blood glucose management. Second, this study stratified the influencing factors of hypoglycemia before constructing and visualizing the risk prediction model, which was conducive to clinical identification of vulnerable T2DM patients, in order to provide early intervention in high-risk groups and reduce the risk of postoperative hypoglycemia. However, this study also has some limitations. For example, we did not find statistical difference in age between the hypoglycemia group and nonhypoglycemia group despite previous studies showing that age appears to be a risk factor of hypoglycemia. It was considered that the limited number of patients included in this study resulted in such nonsignificant difference. Additionally, we did not adequately consider the impact of the type of surgery and the method of anesthesia on the occurrence rate of hypoglycemia.

In summary, by retrospectively analyzing the relevant influencing factors and constructing risk prediction model, as well as developing risk scoring table of postoperative hypoglycemia in T2DM patients during perioperative period, this study has strong clinical practicability in intuitively quantifying the impact of risk factors on outcome variables so as to warning the occurrence of hypoglycemia, thereby adjusting the treatment in time.

## Figures and Tables

**Table 1:** Comparisons of demographics and clinical indicators.

Characteristic	Hypoglycemia group (*n* = 109)	Nonhypoglycemia group (*n* = 331)	*P* value
Sex (male/female) *n* (%)	68 (62.39)/41 (37.61)	204 (61.6)/127 (38.4)	0.888
Age (years)	63.84 ± 10.3	62.26 ± 10.81	0.180
Smoking history (yes)	24 (22.02)	55 (16.6)	0.202
Drinking history (yes)	13 (11.93)	30 (9.1)	0.584
Family history (yes)	50 (45.87)	141 (42.60)	0.550
History of hypertension (yes)	60 (55.05)	186 (56.20)	0.908
BMI (kg/m^2^)	24.00 (21.45, 26.74)	24.68 (22.86, 27.3)	0.007
Duration of diabetes (years)	10 (5, 12.5)	6 (2, 10)	0.002
Preoperative C-peptide (pmol/L)	524.6 (332.1, 858.7)	685.95 (492.98, 937.55)	0.004
Postprandial C-peptide (pmol/L)	1222.5 (685, 2246.75)	1561 (1060, 2483)	0.102
HbA1c (%)	7.8 (6.8, 9.225)	8.4 (7.3, 9.7)	0.043
Preoperative fasting blood glucose (mmol/L)	6.51 (5.10, 9.23)	7.99 (6.66, 10.65)	<0.001
AST (U/L)	17.2 (11.8, 28.2)	20.2 (13.3, 32.8)	0.107
ALT (U/L)	17.3 (14.1, 23.4)	18.6 (14.33, 24.98)	0.673
UA (mmol/L)	312 (246, 393)	326 (254, 399)	0.189
eGFR (ml·min^−1^·(1.73 m^2^)−1)	113 (88.8, 135.5)	116.2 (94.225, 137.9)	0.395
TG (mmol/L)	1.19 (0.8, 1.68)	1.37 (0.98, 2.0775)	0.003
TC (mmol/L)	4.13 (3.25, 4.75)	4.23 (3.415, 5.0575)	0.112
HDL-C (mmol/L)	1.07 (0.86, 1.43)	1.02 (0.83, 1.2375)	0.031
LDL-C (mmol/L)	2.27 ± 0.86	2.44 ± 0.9	0.102
In-hospital blood glucose management			0.017
Department self-management	50 (45.9)	115 (34.7)	
Endocrinology consultation management	36 (33)	99 (29.9)	
Hospital blood glucose management	23 (21.1)	117 (35.3)	
Level of surgery			0.120
I	1 (0.90)	0 (0.00)	
II	3 (2.80)	10 (3.00)	
III	14 (12.80)	66 (20.10)	
IV	91 (83.50)	253 (76.90)	
Incision type			0.172
I	10 (9.30)	37 (11.30)	
II	51 (47.20)	181 (55.20)	
III	47 (43.50)	107 (32.60)	
IV	0 (0.00)	3 (0.90)	
Operation duration (h)	3.58 ± 1.94	3.02 ± 1.79	0.006
Intraoperative blood loss (ml)			0.215
≤200	53 (52.50)	185 (59.50)	
>200	48 (47.50)	126 (40.50)	
Hospital stays (days)	18 (13.5, 23)	16(11, 22)	0.038
Wound healing time (days)	9 (6, 11)	7 (5, 10)	0.002

**Table 2:** Assignments of categorical independent variables.

Variable	Assignment
Preoperative C-peptide (pmol/L)	<500 = 0, ≥500 = 1
In-hospital blood glucose management	Department self-management = 0, endocrinology consultation management = 1, and hospital blood glucose management = 2

**Table 3:** Logistic regression of postoperative hypoglycemia in T2DM patients during the perioperative period.

Factor	*β*	S. E	Wald	*p*	OR (95% CI)
BMI	−0.112	0.042	6.972	0.008	0.894 (0.823, 0.972)
Preoperative FBG	−0.152	0.070	4.717	0.030	0.859 (0.748, 0.985)
Operation time	0.237	0.110	4.619	0.032	1.267 (1.021, 1.573)
Preoperative C-peptide	0.864	0.387	4.969	0.026	2.372 (1.11, 5.069)
Constant	1.647	1.270	1.681	0.195	5.190

**Table 4:** Risk scoring table of postoperative hypoglycemia in T2DM patients during the perioperative period.

Influence factor	Category	Point
BMI	≤18.5	2
	18.6∼23.9	1
	24.0∼27.9	0
	≥28	−2

Operation time	<2	0
	2∼3.9	1
	4∼5.9	2
	6∼7.9	3
	8∼9.9	4
	10∼11.9	5

Preoperative FBG	3.0∼3.9	1
	4.0∼6.0	0
	6.1∼6.9	−1
	≥7.0	−2

Preoperative C-peptide	<500	2
	≥500	0

**Table 5:** The corresponding relation between the total calculated score and predicted probability.

Point total	Estimate of risk
−4	0.0271
−3	0.0428
−2	0.0671
−1	0.1035
0	0.1564
1	0.2296
2	0.3237
3	0.4347
4	0.5527
5	0.6649
6	0.7612
7	0.8366
8	0.8916
9	0.9297
10	0.9646

*Notes*. BMI, body mass index; FBG, fasting blood glucose; AST, aspartate aminotransferase; ALT, alanine aminotransferase; UA, urine acid; eGFR, estimated glomerular filtration rate; TC, total cholesterol; TG, triglyceride; HDL-C, high-density lipoprotein cholesterol; LDL-C, low-density lipoprotein cholesterol; ROC, receiver operating characteristic; AUC, area under the curve.

## Data Availability

The datasets used and/or analyzed during the current study are available from the corresponding author on reasonable request.
